# Ownership of the affected leg is further reduced following deceptive behaviors in body integrity dysphoria

**DOI:** 10.1016/j.isci.2023.107551

**Published:** 2023-08-07

**Authors:** Marina Scattolin, Maria Serena Panasiti, Jasmine T. Ho, Bigna Lenggenhager, Salvatore Maria Aglioti

**Affiliations:** 1 Sapienza University of Rome and CLN^2^S@Sapienza, Italian Institute of Technology, Rome (RM) 00161, Italy; 2 Santa Lucia Foundation, IRCCS, Rome (RM) 00179, Italy; 3 Department of Psychology, Sapienza University of Rome, Rome (RM) 00185, Italy; 4 Department of Psychology, University of Zurich, 8050 Zurich, Switzerland; 5 Department of Psychiatry, Psychotherapy and Psychosomatics, University of Zurich, 8032 Zurich, Switzerland; 6 Department of Psychology, University of Konstanz, 78457 Konstanz, Germany

**Keywords:** Psychiatry, Clinical medicine, Clinical neuroscience

## Abstract

Although predicted by the notion of embodied morality, it remains unknown whether a reduced sense of body ownership (SoO) is associated with increased or decreased dishonesty. To clarify this issue, we tested patients with body integrity dysphoria (BID), a clinical condition characterized by chronic reductions of SoO toward one leg that patients persistently desire to have amputated. Participants with BID played a card game in which they could voluntarily tell the truth or cheat an opponent, and thus either steal or give them money. To assess whether SoO toward the effector limb influences (im)moral decisions, responses were communicated with the affected or the unaffected leg. We found that a higher number of self-gain lies was followed by further reductions of SoO toward the affected leg. Our result supports the idea that reductions of SoO may follow immoral behaviors to distance from unwanted characteristics of the self, like one’s own dishonesty.

## Highlights

• People with body integrity dysphoria (BID) desire the removal of the affected leg
• Ownership and agency are lower for the affected leg than the unaffected one
• Moral behavior is similar for BID and controls and the affected and unaffected leg
• Communicating self-gain lies with the affected leg further decreases ownership

## Graphical abstract

**Figure:**
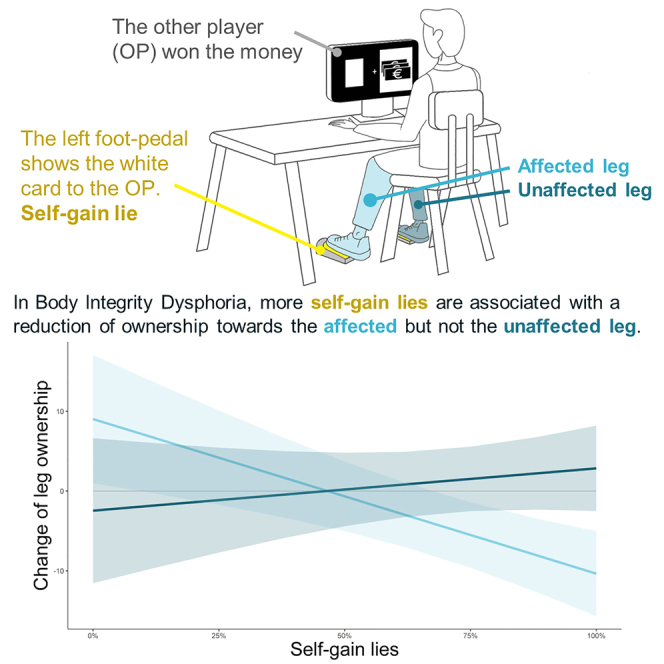

## Introduction

For a long time, researchers considered cognition as amodal and largely independent from sensorimotor information. More recently, embodied cognition approaches have uncovered the interplay between bodily and cognitive functions.^1^ Indeed, some evidence suggests that sensorimotor information contributes to define the meaning of words,^2^ support geometric reasoning,^3^ shape the emotional value associated with memories,^4^ and aid the recognition of the emotional state of others.^5^ The stream of information that continuously reaches the brain is also fundamental for the emergence of higher-order representations of the body.^6^ Information converging from multiple sensorimotor modalities is combined into a unitary perception, the sense of ownership (SoO) for the body, that has been described as one of the key aspects of corporeal awareness (i.e., the conscious, moment-to-moment, organized experience of the body^7^^,^^8^^,^^9^). Such higher-level bodily representations also seem to play a role in cognition. In fact, performance on a fluid intelligence task improves after feeling that a virtual body with a resemblance to Einstein is one’s own,^10^ and experiencing SoO over an avatar of a different skin color or gender can reduce implicit prejudices against people of the same skin color and gender.^11^^,^^12^ All this suggests that sensorimotor information, SoO, and high order mental processes may be strongly associated and could reciprocally influence one another and, as such, a deeper understanding of human behavior may come from unveiling their net of relations.^13^ For instance, both sensorimotor signals and internal representations of the body appear to be involved in the process of decision-making. The somatic marker hypothesis posits that changes of the physiological state of the body support reasoning.^14^ Accordingly, individuals whose autonomic reactivity is reduced make decisions that are less economically advantageous, compared to those with more pronounced activation.^15^ Studies where bodily representations are altered show that these also affect decision-making. In one of these studies,^16^ participants embodied a different-gender or a same-gender virtual body while choosing between a generous option, where both themselves and a known person would receive the same amount of money, and a selfish one, where the participant would get a larger sum but the other person would not be compensated. Participants in a different-gender body made more selfish decisions than those in a same-gender body. This suggests that one’s pre-existing body representation can be modified to include new features, but might feel as a disguise and enable less prosocial decisions. Furthering our understanding of the interconnections between sensorimotor information, SoO and decision-making might be particularly relevant for *immoral* decisions, which come with considerable interpersonal and economic costs.^17^^,^^18^

So far, evidence concerning the link between the body and morality appears somewhat contradictory. On one hand, studies on the role of sensory information suggest that attention to bodily signals may bias decisions toward self-interested choices and judgments. For instance, this has been investigated through Ultimatum Games (UG), where a participant playing as proposer decides how to split a certain sum of money between oneself and another player. The responder can accept the offer and take the money, or reject the offer, in which case neither of the players gets any money. While the rational choice would be for responders to accept any non-zero offer they receive from the proposer, no matter how small, in reality participants tend to refuse offers that they consider unfair, despite their own economic losses.^19^ Interestingly, if participants listen to their own heartbeat or experience painful stimulation during the UG, their choices shift toward their own economic advantage: they offer less to others and accept more unfair offers from proposers.^20^^,^^21^ Sensory signals, like hunger, seem to influence morality in a similar way, favoring choices that increase personal benefits. In fact, the hungrier participants report being, the less they disapprove of other people’s moral choices^22^ and the less honestly they behave.^23^ Overall, this evidence suggests that increased attention to sensory signals and the own body may bias toward more dishonest choices when this is convenient for the self. This effect may be mediated by the value attributed to available stimuli, that highly depends on the homeostatic condition of the body.^24^^,^^25^ This could boost reward-oriented behaviors, and possibly even deceptive decisions, if these profit the self.

On the other hand, investigations of the role of SoO suggest that *low* SoO in healthy participants may be associated with higher immorality. In a recent survey study, participants completed a series of questionnaires that also assessed their SoO and moral identity.^26^ Individuals with a low score on the SoO questionnaire also found moral characteristics to be less relevant for their concept of self, compared to those with high SoO scores. In another study, researchers applied virtual reality to modulate the SoO of participants over a virtual body.^27^ Results showed that the SoO of participants was reduced if they saw their virtual body from an external perspective. Interestingly, when their SoO was reduced, participants also behaved more dishonestly to obtain high (vs. low) rewards. This evidence suggests that a weak SoO may serve the purpose of distancing the self from one’s own negative actions, such as dishonest ones, as in other separation strategies.^28^^,^^29^ For example, by achieving physical separation from negatively connotated stimuli or situations (e.g., by putting a written recollection of negative events within an envelope), individuals manage to reduce the negative emotions associated with the recollected event.^30^ Similarly, people with a history of trauma commonly refer experiencing SoO reductions *during* the traumatic event or when they are reminded of it, as a way to protect the self from the harm that the body went through.^31^ Thus, enhanced SoO may facilitate the association between attributes of the owned body and one’s consideration of the self, while dis-ownership could hinder this association. It derives from this that if a body is not perceived as belonging to the self, its negative attributes (like dishonesty) become less prominent and are less likely to affect one’s identity (as a moral person), which is then preserved. It appears that, at any given time, the SoO and transient changes thereof might influence moral decisions and our daily interactions. However, it is not clear how more permanent alterations of the SoO could impact moral decision-making.

This evidence suggests that despite the role that sensory information has over the emergence of the SoO, their respective modulation may affect morality in different ways. Here, we specifically focused on the effects that long-lasting alterations of SoO have during moral decision-making, when the processing of other bodily signals is intact. These characteristics are typical of the amputation variant of body integrity dysphoria (BID). According to the ICD-11, BID is characterized by an enduring, intense desire to have a physical disability.^32^ In the subtype with amputation desire, individuals with intact sensorimotor processing perceive one or more limbs as not belonging to them, resulting in a strong and persistent desire for limb removal.^33^^,^^34^^,^^35^^,^^36^ Such a sense of disownership toward a part of the body typically originates in adolescence or early childhood^34^ and derives from a sense that the individual’s *physical* body “exceeds” the individual’s *percept* of the body. These symptoms of BID have been associated with structural and functional alterations in areas that are involved in the “body matrix”,^37^ like the primary and secondary somatosensory cortices, the inferior and superior parietal lobules, and the insula.^38^^,^^39^^,^^40^^,^^41^^,^^42^^,^^43^^,^^44^^,^^45^ Given the abovementioned evidence of a link between SoO and decision-making, we set out to investigate whether using parts of the body that are chronically associated with different levels of SoO but intact sensorimotor functioning, like the unaffected and affected limb in BID, could shape the process of moral decision-making.

**Figure 1:**
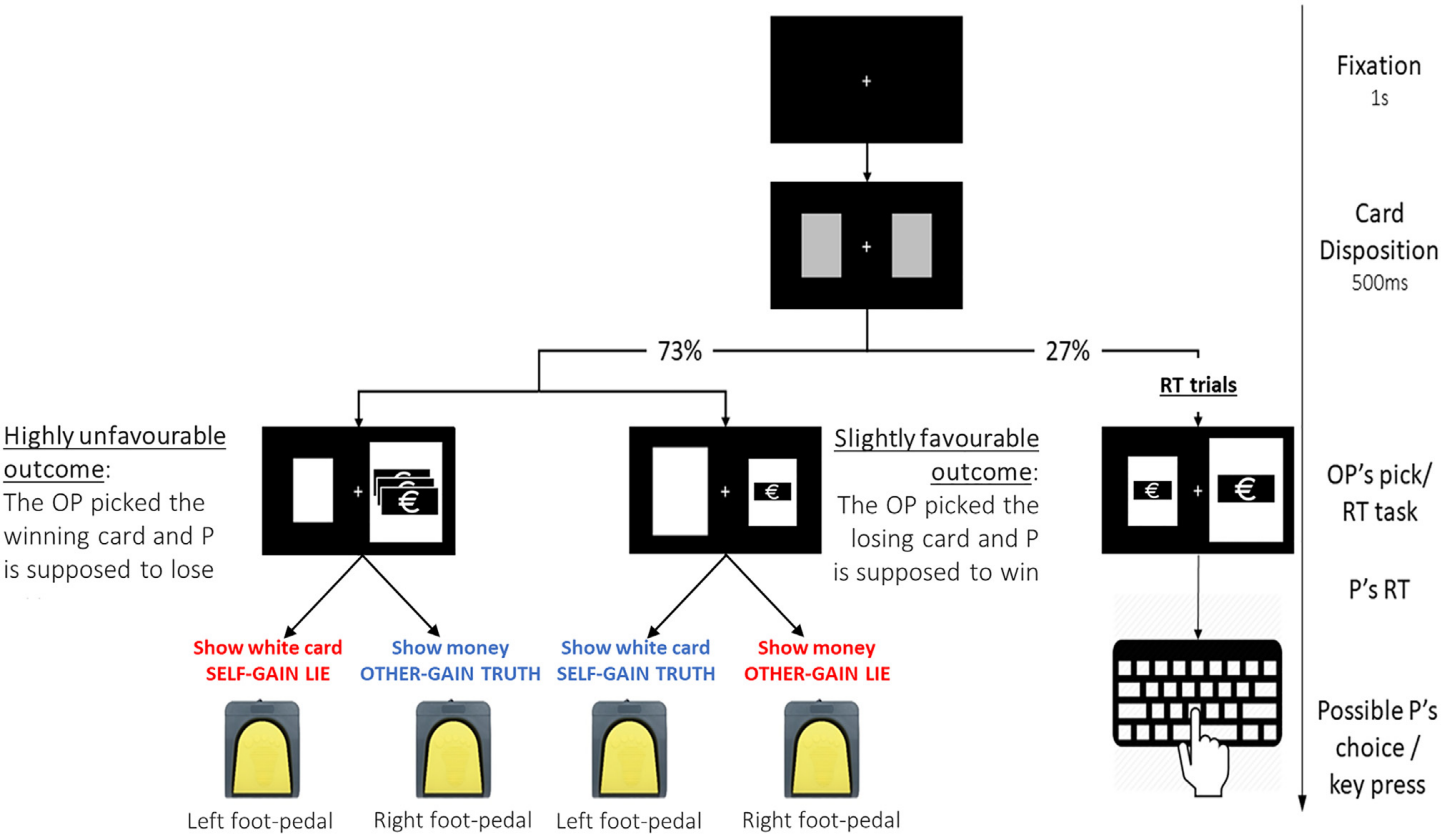
Schematic representation of the unfair version of the Temptation to Lie Card Game (U-TLCG) and of the Reaction Time task (RT task) A timeline of each trial is presented on the rightmost part of the figure. In 73% of the trials, participants (P) play the U-TLCG. This is indicated by the fact that the cards on the screen represent different amounts of money (i.e., one is empty, the other shows one or more banknotes). The other player (OP) has to pick one of the two covered cards. The card that enlarges on the screen represents the OP’s pick. When the OP picks the card representing the money, this indicates that OP won and P lost. This is always associated with the three banknotes card and is therefore tied to a higher reward (*highly unfavorable situation*). When the OP picks the empty card, this indicates that OP lost and P won. These trials are always associated with the card with one banknote, and therefore tied to a lower reward (*slightly favorable situation*). The P can communicate the outcome to the OP, by either showing the empty card or the card with one/three banknote/s. Both appeared on the left (right) side of the screen on half of the trials. By showing the white card, the Ps are communicating that the OP lost (and they won). To communicate that the OP won (and they lost), the Ps show the banknote(s) card. To communicate their decision, Ps were asked to press one of the two foot-pedals, the one on the same side of the card they wanted to show to the OP. To prevent Ps from guessing our hypothesis, we presented a simple RT task as the focus of our investigation. Thus, on 27% of trials, the cards on the screen showed the same amount of money. When this happened, Ps had to press the letter *b* on the keyboard with their dominant index finger. According to this cover story, the U-TLCG trials would help to clarify how much social interactions distract from other tasks.

For this purpose, we compared the behavior of individuals with BID with that of an age-matched group of participants who instead experience similar levels of SoO across their legs. Both groups of participants (BID and Control Group, or CG) completed a modified version of the Temptation to Lie Card Game (TLCG).^46^^,^^47^^,^^48^^,^^49^^,^^50^ In the TLCG, participants play with another person, who repeatedly and blindly picks one among two covered cards. One of the cards comes with a monetary win for the picker, the other with a win for the participant. It is, however, the participant’s job to communicate the outcome of the pick to the other person, who is not given the opportunity of checking this directly. Participants are thus in the position to freely decide whether to lie or communicate the truth to the other player, knowing that their choices will determine their own final payment as well as the other person’s (see Figure 1 for a visual representation of the task). To clarify whether the SoO toward the effector limb could bias participants’ behaviors toward honesty or dishonesty, in each and every trial the participants could provide their response using either of the two available foot-pedals, each placed under one of their feet. Since our BID sample consisted of individuals with a unilateral, lower limb amputation desire, responses made with one of the foot-pedals coincided with the affected leg, while those communicated with the other foot-pedal were made using the unaffected leg. Similarly, participants in the CG were free to use the foot-pedal under their dominant leg or that under the non-dominant one in every trial. Unbeknownst to the participants, we biased the outcomes of the TLCG toward unfairness. Specifically, in the Unfair TLCG (U-TLCG), a win for participants was always associated with the smallest monetary sum, while pickers always won the highest monetary rewards. This bias was motivated by the expected age of individuals with BID (whose mean age ranged from 43.1 to 49.6 years, in studies on BID)^34^^,^^40^^,^^42^^,^^43^^,^^44^^,^^51^^,^^52^^,^^53^^,^^54^^,^^55^^,^^56^ compared to previous TLCG experiments (mean age between 23.1 and 26.7 years).^46^^,^^47^^,^^48^^,^^49^^,^^50^ Most participants in previous TLCG studies were students, who, being generally unemployed, may have been tempted to lie even for small monetary rewards. On the contrary, we expected participants with BID and the age-matched healthy control group to have an income, and to show reduced reward sensitivity compared to younger adults.^57^^,^^58^ Considering that the value of rewards is associated with an increase of cheating behaviors,^59^ we designed the U-TLCG to be more tempting and engaging for older participants. In line with the studies from Scattolin et al.,^26^^,^^27^ we hypothesized that participants with BID might communicate more self-gain lies using their affected leg compared to the non-affected leg, while participants with similar levels of SoO across their legs (those in the CG) might not show the same pattern.

To investigate whether the SoO could change depending on the percentage of self- and other-gain lies communicated with it, all participants used a Visual Analogue Scale (VAS) to rate the statement “How strong is the sensation that your left/right leg is part of your body?”, before and after completing the U-TLCG. We included separate VAS statements to additionally assess the feeling of control over each leg, or Sense of Agency (SoA), another crucial element for the emergence of corporeal awareness.^60^ As the literature does not report any evidence of SoA alteration in addition to those observed for SoO among individuals with BID, we expected to observe similar SoA ratings between our groups and between the affected and unaffected leg of participants with BID.

## Results

### Ownership and agency are reduced for the affected leg

#### SoO ratings

To ensure that the two groups of participants differed in the SoO associated with their lower limbs, SoO ratings collected before the U-TLCG were analyzed through a linear mixed effect model. SoO ratings represented the predicted variable, while group (CG coded as 0, BID coded as 1), leg condition and their interaction term were the fixed predictors. Leg condition was a binary variable and was coded as 0 to indicate the affected leg of participants in the BID group or the non-dominant leg for those in the CG. Oppositely, the unaffected or dominant leg was coded as 1 for participants in the BID and CG groups, respectively. We classified each leg of participants in the CG as either dominant or non-dominant based on individual scores at the footedness subscale of the Lateral Preference Inventory (LPI).^61^^,^^62^ Specifically, the left leg was classified as dominant for participants whose footedness score was below 0; oppositely the dominant leg corresponded to the right one for those whose score was above 0. The distribution of foot preference within each group is reported in Table S1. Participants’ identification number (ID) was included in the model as random intercept.

**Figure 2:**
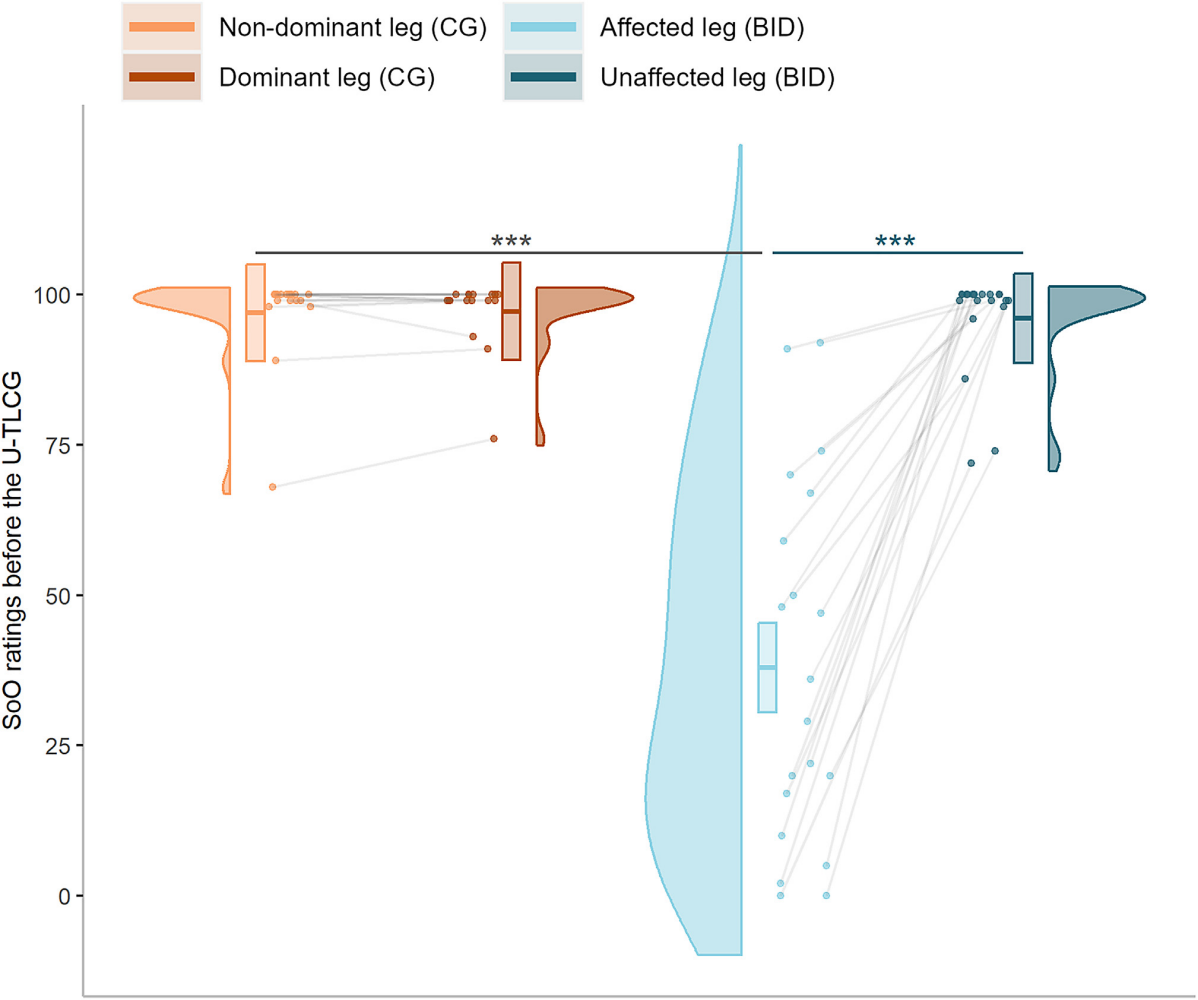
Sense of Ownership (SoO) ratings are reduced for the affected leg Violin plots represent the distribution of SoO ratings at baseline for each leg condition and group of participants. The thick horizontal line within boxes indicates the median, while the lower and upper ends of the boxes represent Quartile_1_ and Quartile_3_, respectively. Connected dots represent how the ratings of the same participant changed across leg condition. Asterisks indicate significance (∗∗∗p < 0.001).

The model (R^2^_marginal_ = 0.71, R^2^_conditional_ = 0.76) shows that factors group (β = −59.05, 95% Confidence Intervals or CI [-70.20, −47.90], *t*(68) = −10.56, p < 0.001) and the interaction between group and limb condition (β = 57.86, 95% CI [43.73, 72.00], *t*(68) = 8.17, p < 0.001) are significant predictors of the SoO toward lower limbs (Figure 2). As expected, post-hoc comparison showed that the dominant and non-dominant leg of control participants did not differ in terms of SoO ratings (estimate = −0.24, 95% CI [-10.81, 10.34], Standard Error or SE = 5.21, t.ratio = −0.05, p = 0.964). A significant difference was observed for participants in the BID group, where the affected leg had lower SoO ratings compared to the unaffected leg (estimate = −58.10, 95% CI [-67.85, −48.35], SE = 4.80, t.ratio = −12.10, p < 0.001). SoO ratings were significantly lower for the affected leg of participants in the BID group than for the non-dominant leg of the CG (estimate = 59.05, 95% CI [47.89, 70.21], SE = 5.59, t.ratio = 10.56, p < 0.001). The SoO ratings of control participants for their dominant leg did not differ from those for the unaffected leg of participants in the BID group (estimate = 1.19, 95% CI [-9.97, 12.34], SE = 5.59, t.ratio = 0.21, p = 0.964). See Table S2 for additional information.

#### SoA ratings

**Figure 3:**
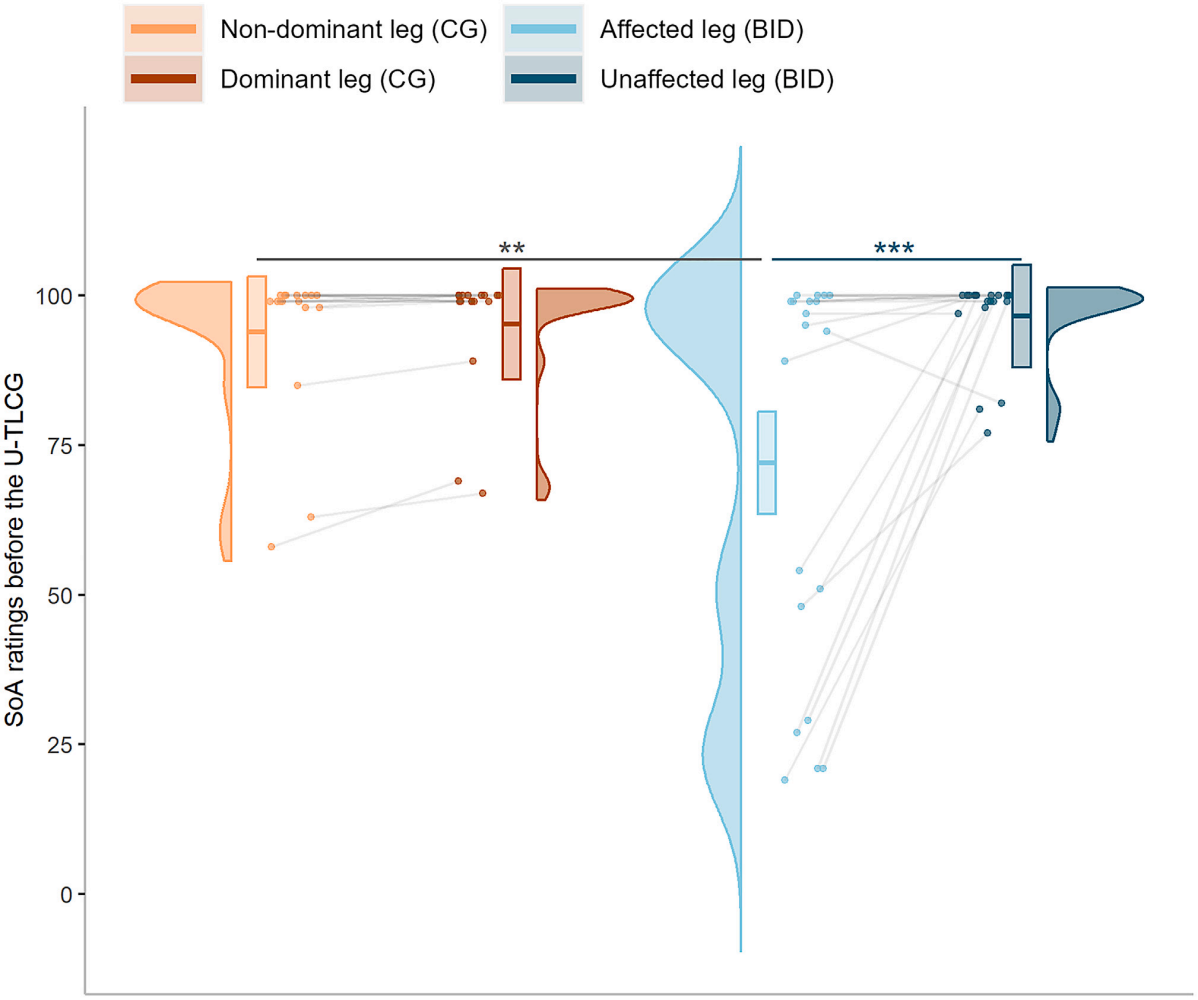
Sense of agency (SoA) ratings are reduced for the affected leg Violin plots represent the distribution of SoA ratings at baseline for each leg condition and group of participants. The thick horizontal line within boxes indicates the median, while the lower and upper ends of the boxes represent Quartile_1_ and Quartile_3_, respectively. Connected dots represent how the ratings of the same participant changed across leg condition. Asterisks indicate significance (∗∗∗p < 0.001).

To verify that participants in the BID and CG groups exclusively differed in the SoO they felt toward their lower limbs, we set SoA ratings as the dependent variable of a linear mixed effect model. Participants’ IDs were included in the model as random intercept factor, while we entered group (CG as 0, BID group as 1), limb condition (the non-dominant or affected leg coded as 0, the dominant or unaffected leg coded as 1), and the interaction between group and limb condition as fixed predictors. Surprisingly, the results of this model (R^2^_marginal_ = 0.22, R^2^_conditional_ = 0.42) showed that factors group (β = −21.89, 95% CI [-34.75, −9.03], *t*(68) = −3.40, p = 0.001) and the group × limb condition interaction (β = 23.21, 95% CI [7.48, 38.94], *t*(68) = 2.94, p = 0.006) were significant predictors of SoA ratings. SoA ratings of participants in the CG did not differ between the non-dominant and the dominant leg (estimate = −1.29, 95% CI [-13.06, 10.47], SE = 5.80, t.ratio = −0.22, p = 0.839; Figure 3). SoA ratings were significantly higher for the unaffected compared to the affected limb of participants in the BID group (estimate = −24.50, 95% CI [-35.35, −13.65], SE = 5.34, t.ratio = −4.59, p < 0.001; Figure 3). SoA ratings for the affected limb of participants with BID were lower than those associated with the non-dominant leg of control participants (estimate = 21.89, 95% CI [9.02, 34.76], SE = 6.45, t.ratio = 3.40, p = 0.002; Figure 3). SoA ratings for the unaffected leg of participants in the BID group were not different from those observed for the dominant leg of control participants (estimate = −1.31, 95% CI [-14.19, 11.56], SE = 6.45, t.ratio = −0.20, p = 0.839; Figure 3). See Table S3.

### Leg ownership does not affect moral decisions

The participants’ mean percentage of lies during the U-TLCG was 34.30% (SD = 20.68%). The percentage of self-gain lies was 56.42% for highly unfavorable trials (SD = 37.80%). Other-gain lies constituted 12.16% of slightly favorable trials (SD = 17.86%).

**Figure 4:**
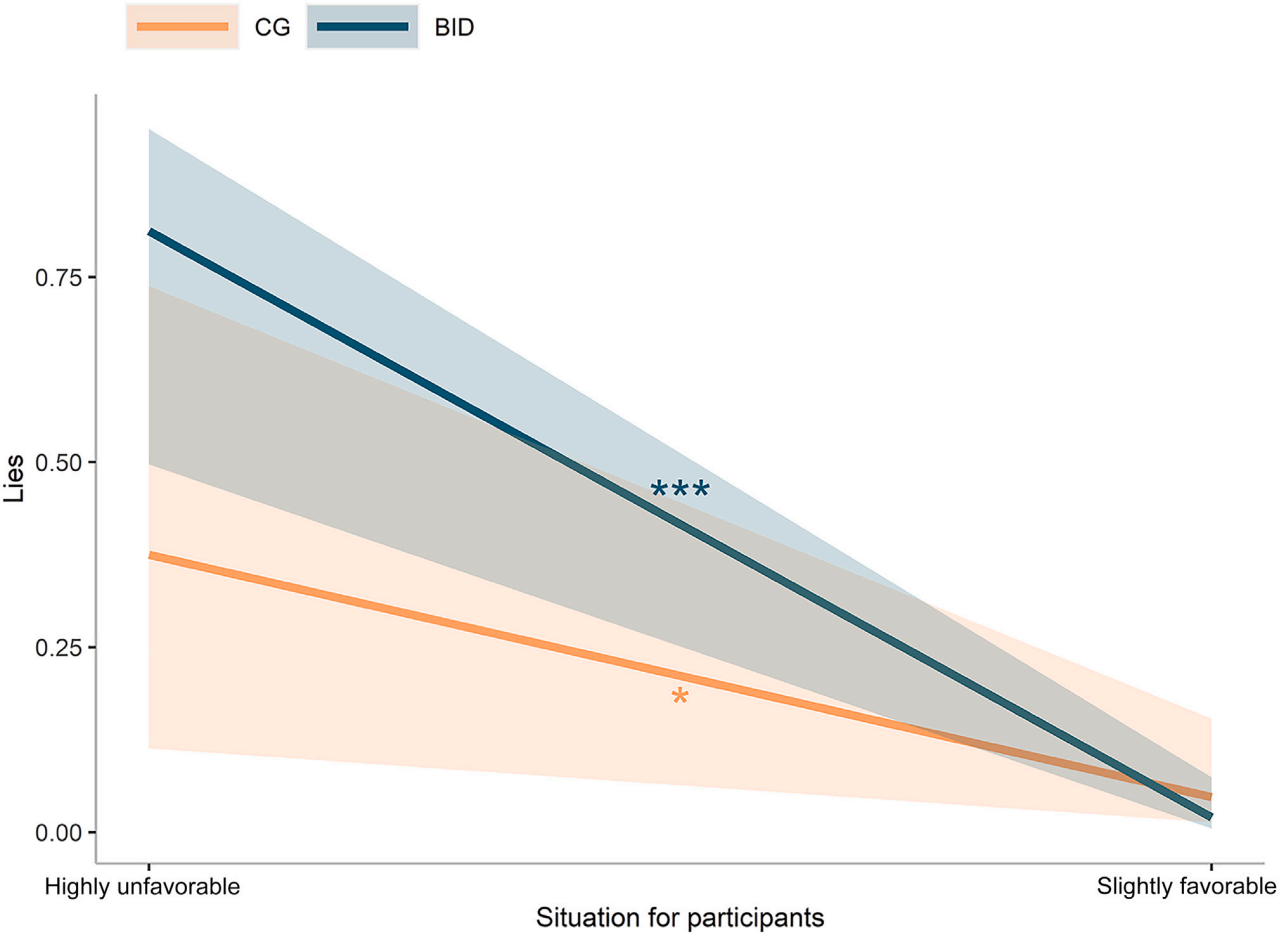
Lies as a function of situation and experimental group The plot represents regression lines for participants with body integrity dysphoria (BID) and those in the control group (CG). The shaded bands represent 95% confidence intervals, and the asterisks indicate significance (∗p < 0.05, ∗∗∗p < 0.001).

Participants’ behavior during the U-TLCG task was analyzed by means of a logistic mixed model. For each trial, a decision to communicate the real outcome was coded as 0, while lies were coded as 1. Factor situation (*highly unfavorable* coded as 0, *slightly favorable* coded as 1) was included as random slope, while participants’ IDs were entered as random intercept factor. To investigate whether the SoO toward the limb that the participants used to communicate each outcome could predict their choices, leg condition (0 for non-dominant and affected leg, 1 for dominant or unaffected leg) was set as fixed factor together with situation (*highly unfavorable* as 0, *slightly favorable* as 1), group (CG coded as 0, BID coded as 1) and all their interactions. To control for the possible role of the SoA associated with each limb and for how engaging the task felt to participants, baseline ratings of SoA and involvement in the U-TLCG were included as covariates. This analysis (R^2^_marginal_ = 0.33, R^2^_conditional_ = 0.78) shows that the situation significantly predicted behavior in the task (β = −2.48, 95% CI [-4.50, −0.46], *z* = −2.41, p = 0.016), as did the interaction between situation and group (β = −2.87, 95% CI [-5.69, −0.05], *z* = −1.99, p = 0.046). Post-hoc comparisons revealed that both groups of participants lied more in highly unfavorable trials compared to slightly favorable ones (CG: estimate = 2.62, 95% CI [0.69, 4.56], SE = 0.99, z.ratio = 2.66, p = 0.016; BID: estimate = 5.38, 95% CI [3.51, 7.24], SE = 0.95, z.ratio = 5.66, p < 0.001, see Figure 4), and that this difference was larger in the BID than in the CG group (estimate = −2.75, 95% CI [-5.44, −0.07], SE = 1.37, z.ratio = −2.01, p = 0.045). Additionally, the two groups of participants differed more in terms of self-gain lies compared to other-gain ones (estimate = −2.75, 95% CI [-5.44, −0.07], SE = 1.37, z.ratio = −2.01, p = 0.045), although the difference between the two groups remained non-significant in both situations (highly unfavorable: estimate = −2.07, 95% CI [-4.13, −0.002], SE = 1.05, z.ratio = −1.96, p = 0.066; slightly favorable: estimate = 0.69, 95% CI [-1.04, 2.42], SE = 0.88, z.ratio = 0.78, p = 0.436).

No other predictor was significant (see Table S4).

### Self-gain lies are followed by further reductions of leg ownership

To investigate whether the SoO associated with each leg could change depending on the percentage of self-gain lies communicated with that specific leg, we ran a linear mixed effect regression model. In this model, the difference between SoO ratings before and after the U-TLCG was included as dependent variable, while we set participants’ IDs as the random intercept. We included group (CG as 0 and BID as 1), leg condition (non-dominant leg of the participants in the CG group or affected leg of the participants in the BID group were coded as 0, the dominant or unaffected leg as 1) and the percentage of self-gain lies communicated with each leg as fixed predictors. We entered the change in SoA ratings for each leg and the participants’ involvement in the task as covariates.

**Figure 5:**
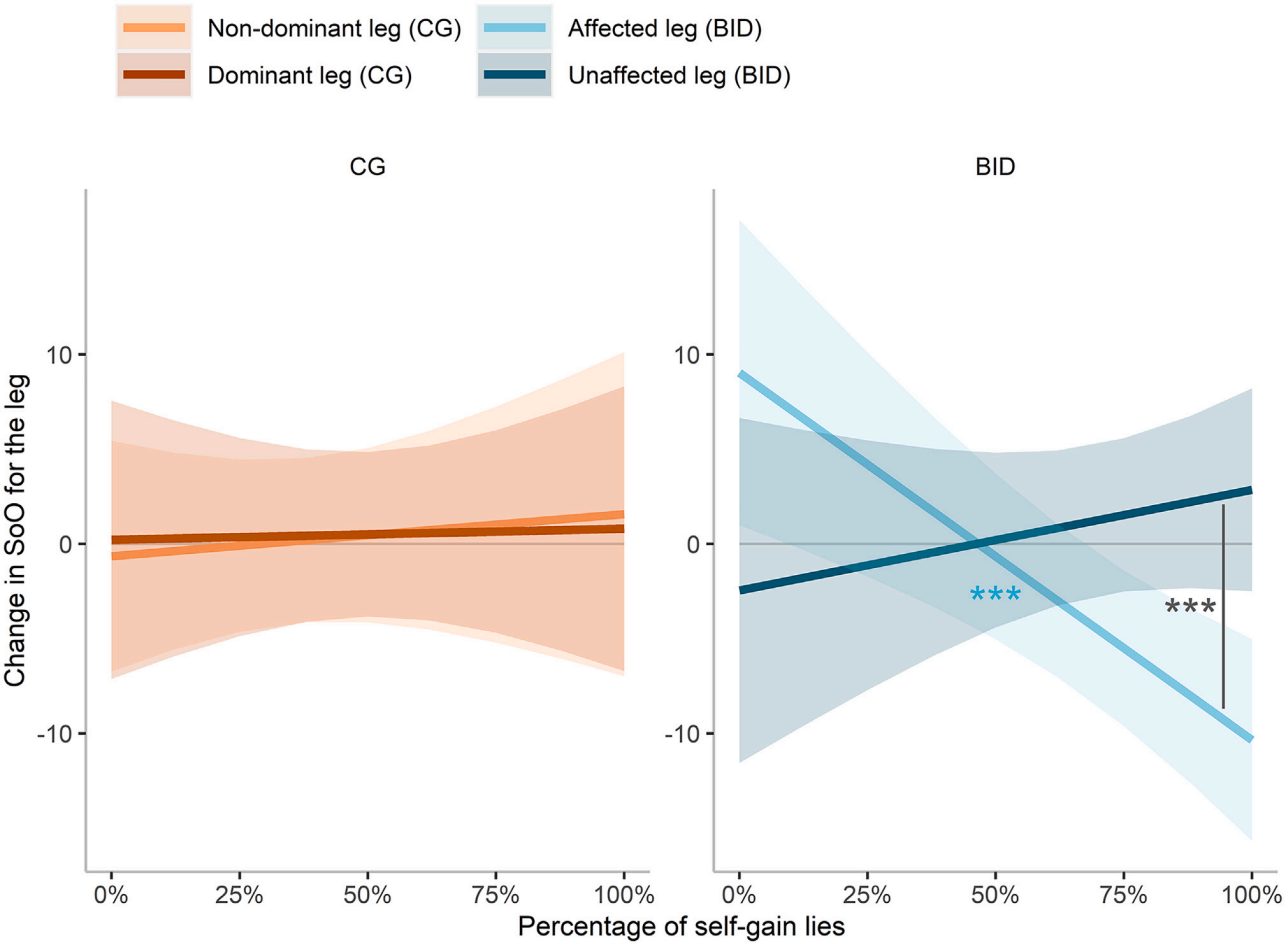
Self-gain lies during the Unfair Temptation to Lie Card Game (U-TLCG) are followed by further reductions of the sense of ownership (SoO) associated with the affected leg The leftmost panel represents the change of SoO ratings for participants in the control group (CG), while ratings of participants in the body integrity dysphoria (BID) group are displayed in the rightmost panel. The plot shows regression lines for each leg. The horizontal line at 0 indicates the absence of difference between the two assessments. Values above the horizontal line indicate that SoO ratings are higher following the U-TLCG. Values below the horizontal line indicate that SoO ratings were lower after the U-TLCG. The shaded bands represent 95% confidence intervals. Asterisks indicate significance (∗∗∗p < 0.001).

In this model (R^2^_marginal_ = 0.24, R^2^_conditional_ = 0.35), our participants’ involvement during the U-TLCG significantly predicted a change in SoO over time (β = −2.38, 95% CI [-4.68, −0.08], *t*(62) = −2.07, p = 0.043). The interaction between group and percentage of self-gain lies (β = −21.61, 95% CI [-37.60, −5.63], *t*(62) = −2.70, p = 0.009) and the interaction between group, leg condition and percentage of self-gain lies (β = 26.33, 95% CI [4.54, 48.13], *t*(62) = 2.42, p = 0.019) were also significant. Post-hoc comparisons on the 3-way interaction revealed that, in the BID group, as the percentage of self-gain lies increased, the SoO for the affected leg significantly decreased after the U-TLCG (estimate = −19.39, 95% CI [-29.87, −8.91], SE = 5.35, t.value = −3.63, p < 0.001; Figure 5). However, the percentage of self-gain lies was not associated with a significant change of the SoO for their unaffected leg, after the U-TLCG (estimate = 5.31, 95% CI [-6.43, 17.05], SE = 5.99, t.value = 0.89, p = 0.379; Figure 5). At high percentages of self-gain lies, SoO ratings for the affected leg of the BID participants decreased more than those associated with their unaffected leg (estimate = 12.07, 95% CI [5.51, 18.63], t.value = 3.61, p < 0.001, Figure 5). Further information regarding the results of this regression model are reported in Table S5.

### Other-gain lies do not modulate leg ownership

To clarify whether the percentage of other-gain lies could predict a change of the SoO, we computed a linear mixed effect regression model including the fixed predictors group (0 for participants in the CG and 1 for those in BID group), limb condition (coded as 0 for the non-dominant and affected leg of participants in the CG and BID groups, respectively, and as 1 for the dominant and unaffected leg) and percentage of other-gain lies communicated with each leg. The change in SoO ratings was the dependent variable, while the change in the SoA toward each leg and the ratings of involvement in the U-TLCG were the fixed covariates. We set participants’ IDs as random intercept.

Results of this analysis (R^2^_marginal_ = 0.09, R^2^_conditional_ = 0.14) showed that none of the fixed factors could significantly predict a change in SoO ratings (Table S6; Figure S1).

### Leg agency is not affected by self- or other-gain lies

To investigate whether the percentage of self- or other-gain lies in the U-TLCG could predict a change in the SoA associated with each leg, we computed two separate linear mixed effect models. These included the change in SoA ratings from before to after the U-TLCG as dependent variable, while we set group (0 for participants in the CG and 1 for participants in the BID group), the percentage of self- or other-gain lies (one in each model) communicated with a specific leg, and limb condition (0 for the non-dominant and affected leg and 1 for the dominant or unaffected leg) as fixed predictors. Both models included the change of SoO for each limb and the participants’ involvement in the task as fixed covariates. Participants’ IDs were used as random intercept.

Results suggest that none of the predictors was significantly associated with a change in SoA ratings, for either of the two models (see Tables S7 and S8).

## Discussion

In the present study, we investigated the extent to which chronically altered corporeal awareness (i.e., a lower SoO) influences moral decision making and viceversa. We compared individuals who experience dis-ownership of one leg (the BID group) with a matched group of healthy participants (CG group). Both groups completed the unfair version of the TLCG (U-TLCG) in which they were tempted to lie for high rewards. To clarify whether the SoO associated with each leg could bias individuals’ choices toward a higher or lower number of self-gain behaviors, participants communicated their decisions using either the unaffected or the affected leg (or, for the CG, the dominant or non-dominant leg).

Our findings show that the participants lied more to keep the money to themselves (i.e., in the highly unfavorable situations) than to give the money to the other person (i.e., in the slightly favorable situations). These results mirror those found in the original version of the TLCG.^47^ Notably, in the present study, unfavorable conditions were always associated with high rewards, therefore adding to the notion that opportunities for higher payoffs favor dishonesty.^59^ However, the number of lies was not predicted by the leg that the participants used to communicate their decisions, nor did it differ between the two experimental groups.

While these results suggest that long-term reductions of the SoO do not modulate moral behavior, at least in this specific task, it may be that behavior during the task did modulate the SoO for each limb. In fact, after completing the U-TLCG, participants in the BID group showed a significant further reduction of their SoO for the affected leg, that was significantly associated with the number of self-gain lies communicated with the same leg. Specifically, we observed that the higher the percentage of self-gain lies, the more the SoO toward the affected leg decreased. This suggests that even in BID, distancing from one’s body might be a way to also distance oneself from negative attributes and events.^29^ Accordingly, a recent study found that thermal stimuli are perceived as less painful when delivered to the affected (vs. unaffected) leg of participants with BID.^52^ The further reduction of SoO observed here could represent a strategy to separate from the negative behaviors that participants with BID enacted with the affected leg. In fact, individuals aim at maintaining a positive concept of self, even in the face of their own moral violations.^63^ To balance a moral self-concept with their own immoral deeds, individuals can use a variety of mechanisms, such as cleansing behaviors^64^ or other embodied procedures that elicit a sense of physical or mental separation between the self and negative attributes.^28^ Crucially, the SoO is not a fixed trait but rather a fluent representation originated by the continuous integration of signals reaching the brain.^7^ This malleability is what allows whole, external bodies,^65^ body parts^66^^,^^67^ and even objects^6^ to feel as if they belong to oneself, under specific circumstances.^8^ For the same reasons, one’s own body and parts thereof may feel more or less like oneself across time. Thus, it is possible that when the affected leg is used to communicate a self-gain lie, this negative feature, i.e., dishonesty, is added to the ones already associated with the leg (e.g., not matching the body image, over-completeness, being in the wrong body because of the affected leg)^34^ and can sharpen the desire to distance oneself from it. The results presented here somewhat resemble those of other studies, which also highlighted an association between dishonesty and reduced SoO.^27^

It may be argued that, if SoO serves the purpose of disconnecting the self from negative features, it may also be employed to achieve connection with positive ones. In the case of this study, other-gain lies could have predicted an increase of the SoO for the legs. However, we found that this type of lies did not modulate the SoO for the affected leg. This could be due to the double-faced nature of other-gain lies, which, although associated with more positive attributes, like altruism, remain deontologically immoral.

In contrast to our observations for the affected limb, the SoO toward the unaffected leg was not predicted by how often participants in the BID group had lied for a self-gain. Indeed, the percentage of lies that they communicated with the unaffected leg was not associated with a change of SoO after our task. This result may be due to a more malleable SoO toward the affected limb. In fact, individuals with BID experience limb ownership illusions more vividly than controls, and this was found to correlate with the strength of their desire for amputation.^53^ Similarly, their SoO toward the affected limb appears to rely more strongly on momentarily available visuotactile information, compared to the unaffected limb.^35^ Therefore, it is possible that a highly malleable SoO allows individuals with BID to more easily distance from the negative attributes of their affected leg, to protect their higher-level self-image. In line with this, also the participants in our CG group did not show significant changes concerning the SoO, nor were these observed following dishonest behaviors.

This result could also be observed from an opposite perspective and interpreted as *a lack of* SoO decrease for the *unaffected* leg. In other words, this may be interpreted as evidence that using the unaffected leg for a high number of self-gain behaviors may prevent a reduction of SoO. However, considering that (1) SoO is more malleable for the affected (vs. unaffected) leg,^35^^,^^53^ and that (2) an increase of self-gain lies during the U-TLCG was associated with a further decrease of SoO toward the affected leg, but not the unaffected one (Figure 5), we argue that the difference observed at higher percentages of self-gain behaviors reflect a modulation of SoO for the affected leg.

This evidence indicates that when SoO is more malleable, as in BID,^35^^,^^53^ it could be more easily reduced to elicit a sense of separation from recent, dishonest actions. At the same time, our data suggest that when the SoO is less stable, the circumstances we’re in could more strongly affect our moral decisions. In fact, both groups of participants lied more for self-gains (vs. the gain of others), but we found that this tendency is stronger in BID. It is possible that because participants with BID could more easily rely on SoO changes to distance from their own negative behavior, this could also allow their decisions to be based more on situational factors than on rigid, deontological, moral standards. However, we want to stress again here that the participants in the two groups lied to similar extents. Thus, neither a malleable SoO nor BID symptomatology should be associated with a more dishonest conduct.

We also found that, in addition to the reduction of SoO, participants in our BID group displayed significantly lower levels of explicit SoA for the affected limb compared to the unaffected one. To our knowledge, the SoA for the affected as compared to the unaffected limb has not yet been systematically addressed. This is surprising, as both the SoO and the SoA have been described to be core aspects of corporeal awareness.^68^^,^^69^ Thus, it might be argued that our results reflect differences in the sense of control associated with the unwanted limb, rather than alterations of SoO. We tried to answer this question by including our SoA measure in all relevant models. The results of these analyses show that SoA ratings cannot significantly predict the behavior in the task, and that the percentage of lies communicated with each limb is not associated with changes of SoA. While these data suggest that explicit SoA is not better than SoO in predicting our participant’s behavior, we are aware that explicit ratings only capture one component of SoA, leaving out other important facets. In fact, some argue that implicit measures of SoA, like intentional binding and sensory attenuation, are more reliable methods of assessments, as they are less affected by individual beliefs and biases.^68^ Thus, implicit measures of SoA may confirm whether BID symptomatology involves further, localized SoA alterations, and clarify whether these can affect the process behind (im)moral decisions. Future studies should look into these facets of BID symptomatology.

Our results provide additional support for the role of SoO as a distancing mechanism from negative deeds. More specifically, this evidence shows that reductions of the SoO may occur *after* dishonest behaviors, as an alternative way to separate the self from immorality. Such results offer relevant insights into possible, novel ways of coping with the strong amputation desire of BID. If reductions of the SoO for the limb follows immoral behaviors, enhanced embodiment may be observed after positively connotated actions. We argue that future studies on BID should aim at clarifying this hypothesized association and its temporal duration. If such an association is in fact in place, individuals with BID might benefit from the use of footswitches during online communications, especially when these are perceived as positive. Similarly, collaborative games may have analogous benefits if these can be played using foot keys. However, we stress that these should be regarded as strategies that individuals independently choose to employ, if alleviating the negative feelings associated with a disowned and affected leg is something they wish to achieve.

### Limitations of the study

While this study aimed specifically at investigating the implications of one of the core features of BID, that is, SoO reductions, other facets remain poorly understood. In fact, BID is a very complex and heterogeneous condition, whose different symptomatology could be associated with specific cognitive and personality traits. Future investigations into the behavior of individuals with BID should consider assessing whether possible differences can be (partially) ascribed to specific cognitive and personality traits.

That BID symptomatology is not limited to chronic reductions of SoO is suggested by our data, which surprisingly show that SoA could also be reduced in individuals with BID. The results presented here suggest that SoA alterations may not be linked to moral decision-making. These, however, are not conclusive as our assessment of SoA is limited to explicit SoA judgments, leaving out other facets. In fact, SoA is a very complex experience that arises from the integration of motor and non-motor cues and which is characterized by both explicit and implicit components.^68^ Assessment of the latter relies on perceptual distortions associated with the experience of agency, like perceiving intervals between action and outcomes as shorter than they actually are (intentional binding^70^) or sensory outcomes as less intense if caused by oneself (sensory attenuation^71^). In the case of this study, implicit measures could have clarified whether the SoA alterations we observed in the BID group are limited to, or go beyond, agency *judgments*. One could hypothesize that weaker perceptual distortions may be observed when actions like pressing a foot-pedal are executed with the affected (vs. unaffected) leg, which would suggest that modulations of SoA can be limited to part of the body. Considering the involvement of SoO and SoA in BID symptomatology, future studies should aim at clarifying whether the reduction of SoO that individuals with BID feel toward the affected leg extends also to the SoO *for the movement* (the feeling that body movements are mine but not necessarily determined by me).^72^^,^^73^^,^^74^

Crucially, one limitation of the present study is that, by always associating high and low rewards with the unfavorable and favorable situation, respectively, we cannot make any inference regarding the modulating role of rewards. Our choice was motivated by the age of our sample; however, this aspect necessitates further investigation in future studies. For example, we may hypothesize that participants in the BID group may want to distance themselves more from lies associated with high rather than low payoffs for the self.

## STAR★Methods

### Key resources table

**Table:** 

REAGENT or RESOURCE	SOURCE	IDENTIFIER
**Deposited data**		

Data from 37 human participants and code for replication of analyses	This paper; Mendeley Data	Mendeley Data: https://doi.org/10.17632/trw6wdpd2r.1

**Software and algorithms**		

E-Prime (version 2.0)	Psychology Software Tool	https://pstnet.com/products/e-prime/
PsyToolkit (version 2.6.1)	Stoet et al.,^75^	https://www.psytoolkit.org/
R (version 4.2.2)	R Core Team^76^	https://www.R-project.org/
RStudio (version 2022.12.0.353)	Posit team^77^	https://posit.co/products/open-source/rstudio/

### Resource availability

#### Lead contact

Further information and requests should be directed to and will be fulfilled by the lead contact, Marina Scattolin (marina.scattolin@iit.it).

#### Materials availability

This study did not generate new unique materials.

### Experimental model and study participant details

#### Participants

Considering the rarity of BID and the secrecy that often comes with this condition,^34^ we recruited a sample of 20 volunteers (females = 5, mean age = 46.55, standard deviation or SD = 12.13) who desired the amputation of one of their lower limbs. Of these participants, 11 (55%) desired amputation of the left leg; for the remaining 9 participants, this desire concerned the right leg (45%). In line with other studies,^34^ the desire for amputation first emerged in childhood and early adolescence (between the ages of 6 and 12). None of the participants in this group underwent amputation prior to the study. The amputation desire of participants in the BID group was assessed using an adapted version of the Zurich Xenomelia Scale (ZXS).^51^ In this version, the participants’ agreement with each of the 12 items of the questionnaire was rated along a Visual Analogue Scale (VAS). The VAS scale ranged from “clearly does not apply” (scored as 0) to “clearly applies” (scored as 100). Participants in the BID group showed a mean score of 84.99 (SD = 13.72) out of 100 in the Pure Amputation Desire subscale of the ZXS. None of these participants reported being diagnosed with a neurological disorder. However, two volunteers (10%) in the BID group reported a history of psychiatric disorders and one of them (5% of the BID sample) was taking antidepressant medication at the time of testing. When they took part in this study, seven other participants (35% of the BID group) were using medications for the treatment of non-psychiatric conditions. The control group (CG) is composed of 17, aged-matched participants (females = 7, mean age = 46.24, SD = 11.59) who had not undergone nor desired lower limb amputation. One participant in the CG (5.88%) reported a diagnosis of psychiatric disorder and being undergoing drug treatment when tested for this study. Six other volunteers (35.20% of the CG) were following different types of medical treatment at the time of participation. A more detailed anamnesis of participants in each group can be found in Table S9.

All participants provided written informed consent to participation in the study and granted permission to the use of their data. At the end of the experimental session and after reading a written debriefing form, all participants confirmed their authorization to use the data collected during the study.

### Method details

#### Materials

The participants completed all parts of this study while sitting on a chair in front of a table. We used the online version of PsyToolkit (version 2.6.1)^75^ to program and administer all the questionnaires included in the study. All parts of the U-TLCG were programmed and presented to the participants by means of E-Prime 2.0 (Psychology Software Tool, PA). The participants completed all questionnaires and the U-TLCG using a 15-inch laptop positioned at approximately 60 cm from their eyes. During the U-TLCG, the participants provided their responses via two foot-pedals, one under each foot (MagiDeal SZADKJ0002).

#### Unfair Temptation to Lie Card Game (U-TLCG)

The classic Temptation to Lie Card Game (TLCG)^46^^,^^47^^,^^48^^,^^49^^,^^50^ was adapted to the purposes of this study. To investigate whether the SoO associated with each leg could predict the tendency to lie or refrain from lying when this entailed using that leg, we asked participants to provide their preferred responses by means of foot pedals. However, and in order to prevent participants in the BID group from guessing our hypothesis, we introduced a simple Reaction Time task (RT task) and a cover story. All participants were told that the aim of the study was to investigate the extent to which social interactions could distract people from other activities. Thus, participants were persuaded that the main task was the RT task. More specifically, participants were informed that the screen in front of them would display cards depicting different amounts of money (that is, one banknote, three banknotes or no banknote) but that, in each trial, only two cards would be shown. In part of the trials (12 out of 44), the two cards on the screen would be identical. Whenever this happened, participants pressed the letter *b* on the keyboard as quickly as possible, using their dominant index finger. In the remaining trials, cards differed from one another, and participants had to follow different instructions. We told participants that trials where the two cards differed were part of a game that they would complete with another player. The game involved two roles and while the other player had to select one of two covered cards, participants were asked to communicate the outcome of the other player’s picks (that is, whether the other person had won or lost). Crucially, and unbeknownst to participants, the other person’s picks were generated by a computer. To cover for the other person’s absence, participants were told that the other player had been tested in previous days and had been assigned the role of picker. To fill this role, the other person had been instructed to pick one out of two covered cards: one of the two cards was always empty, while the other always displayed money. Money could be represented as one banknote or as three banknotes. However, we told participants that the picker was not given the possibility of seeing the picked cards, rather, all draws had been recorded and were shown to the participants themselves. In this version of the TLCG, the participants saw one card enlarge on the screen. This was the card that the other player drew. When the selected card showed either one or three banknotes, it indicated that the other player had picked the winning card and thus the participant had lost (*Unfavorable situation* for the participant). Else, if the white card enlarged, it meant that the other player had picked the losing card, and therefore the participant had won (*Favorable situation* for the participant). Because the participants informed the picker of each outcome, they had the opportunity of lying to change the game outcome. In fact, both the participants and the other player would be paid according to the communicated outcome, and not on the basis of the actual pick. To communicate the outcome of their choice, participants were instructed to select the card they wanted to show to the picker using one of the two foot pedals under their feet. Pressing the right pedal (under their right foot) indicated that participants wanted to show the card displayed on the right-side of the screen, pressing the left pedal (under their left foot) indicated that the left-side card should be shown to the other player. The card representing money was presented on the right of the screen on 50% of trials, while it was displayed on the left side on the remaining 50%. Also, participants could choose their response among four possibilities: an *other-gain truth* (the other person won and participants told the truth), a *self-gain truth* (the other person lost and participants told the truth), a *self-gain lie* (the other person won and participants lied), or an *other-gain lie* (the other person lost and participants lied). To make the TLCG task more tempting for older participants, *unfavorable* trials were consistently associated with *high rewards* (*Highly unfavorable* condition) while *favorable* trials always entailed a *low reward* win (*Slightly favorable* condition). This manipulation put participants at a disadvantage: if they were to always tell the truth, their final gains would be lower than those of the other player. Crucially, participants were never made aware of this unfair manipulation.

For a schematic representation of the task, see Figure 1.

#### SoO and SoA ratings

Prior to the experiment, the SoO for each of the legs (and arms) was assessed using a VAS scale to rate the following statement: “How strong is the sensation that your left/right arm/leg is part of your body?”. The VAS scale ranged from 0 (labeled as “not part of my body”) to 100 (labeled as “fully part of my body”). To investigate whether behavior to the U-TLCG could influence participants’ SoO over their lower limbs, the same statements were rated after completion of the task. To confirm that the levels of SoA toward the limbs are similar across legs and groups, specific VAS ratings were obtained to measure the feelings of control over each limb. In this case, statements were phrased as follows: “How strong is the sensation that the movements of your left/right arm/leg are controlled by you?”. VAS scales measuring SoA ranged from 0 (“not controlled by me”) to 100 (“fully controlled by me”). SoA ratings were collected at the beginning of the study and then after the U-TLCG, together with SoO ratings.

The order of statements assessing SoO and SoA was randomized in both presentations.

To understand whether behavior in the U-TLCG could predict changes in SoO and SoA toward each leg, we calculated how much the ratings changed from one assessment to the following one. Specifically, we subtracted participants’ ratings of SoO and SoA *before* the U-TLCG from the corresponding ratings *after* the U-TLCG. This was computed separately for each leg. Positive values indicated an increase in the SoO or the SoA for a specific leg. Negative values indicated that, following the U-TLCG, there was a decrease in the SoO or SoA associated with the leg.

#### Procedure

Participants were met at the entrance of the building and accompanied to the room where this and other experiments took place. The current study constituted part of a larger project with the overarching goal of investigating different aspects of bodily self-consciousness by comparing data from individuals with and without BID desires. Therefore, participants received written information regarding all experiments that they would take part in. All participants signed the informed consent, confirming their willingness to take part in all studies and providing permission to use their data. Participants began the testing session by completing a first set of questionnaires. In addition to general, demographic information (i.e., sex, age in years, highest education degree received), participants were asked to report possible neurologic and psychiatric conditions and whether they were under medication treatment at the time of testing. Methods described in Adler et al. (2000)^78^ and Ostrove et al. (2000)^79^ were adapted to collect a measure of participants’ subjective economic status (SES). Specifically, participants were shown a horizontal VAS scale going from 0 (labeled as “People who have the least money”) to 100 (labeled as “People who have the most money”) and were asked to position themselves along this continuum. While demographic questions were followed by SoO and SoA ratings for participants in the CG, participants in the BID group filled the Zurich Xenomelia Scale (ZXS)^51^ before rating the SoO and SoA associated with each limb. Following this, all participants were asked to complete the Handedness and Footedness subscales of the LPI.^61^^,^^62^ The Footedness subscale (which presents items like “With which foot would you kick a ball to hit a target?”) was included to assess foot preference and identify the dominant leg of each participant, especially those in the CG. However, the purpose of items assessing handedness (like, for example, “With which hand do you draw?”) was that of supporting the cover story used in the U-TLCG. In line with this, the experimenter checked participants’ handedness subscale scores before the U-TLCG. Scores below 0 indicated a preference for the left hand, while scores above 0 suggested a preference for the right one. Based on this score, participants were instructed to use the left or right index finger throughout the U-TLCG. The distribution of left/right hand and foot preference within each group is reported in Table S1. After completing this first set of questionnaires and before the U-TLCG, participants in the BID group completed one of the other studies included in this project. Then, all participants sat in front of a computer. They were instructed to place one foot over each pedal and their dominant index finger over the letter *b* of the keyboard. This position was to be maintained for the entire duration of the experiment. Instructions of the U-TLCG were then displayed on the screen and readout by the experimenter. Instructions were followed by 23 practice trials, during which proper understanding of the task instructions was ensured. Upon completion of the U-TLCG, participants again rated their SoO and SoA toward each limb. Then, they completed a manipulation check questionnaire, which assessed how engaged they were in the task. As in Panasiti et al. (2016)^49^ and Schepisi et al. (2020),^50^ we focused on the question “How involved did you feel in the game?”, to which the participants answered using a 5-point Likert scale (from 1, “Not at all”, to 5, “Very much”). The involvement ratings of participants in the BID group (mean = 3.75, SD = 1.07) did not differ from those of the CG (mean = 3.53, SD = 1.01) (*t*(34.60) = −0.65, 95% CI [-0.92, 0.47], p = 0.523).

At the end of the entire testing session, a written debriefing form was provided, which informed participants that they had played the U-TLCG against the computer and revealed the real purposes of this experiment. Permission to use the data collected during the study was confirmed in writing by all participants.

All methods and procedures of this study were reviewed and approved by the Cantonal Ethics Committee of Zurich (BASEC-Nr. 2018-01699). We did not assess the ethnicity and ancestry of participants who took part in this study.

### Quantification and statistical analysis

Data collection was carried out from January 2020 to January 2021 at the University of Zurich (CH), where a total of 40 participants took part in this study. We recruited 20 participants who desired but had not undergone amputation of one of their lower limbs (BID group). An equal number of age- and gender-matched individuals without this desire constituted the control group (CG). Of the 20 volunteers in the CG, one did not complete the U-TLCG and two other participants showed no foot preference, which prevented the distinction between their dominant and non-dominant leg. For these reasons, all three participants were excluded from further analyses.

We used RStudio software (version 2022.12.0.353)^77^ to handle and analyze all data in R (version 4.2.2).^76^ Mixed effects models were computed with package *lme4* (version 1.1–31). Specifically, we used function *lmer* to apply linear mixed effect modeling to continuous dependent variables (i.e., SoO and SoA ratings at baseline and their change after playing the U-TLCG), and function *glmer* for logistic mixed effect modeling of binomial dependent variables (i.e., the decision to lie or tell the truth during the U-TLCG). We dealt with the nonindependence of our observations by including participants’ IDs as random intercept in all mixed-effects models. To account for the different effect that our experimental manipulation may have over different participants, we modeled factor *Situation* (*highly unfavorable* or *slightly favorable*) as fixed *and* random slope factor of the logistic mixed effect model.^80^ Alpha was set at 0.05. Details about each model can be found in the results sections, Figures 2, 3, 4, 5, Tables S2–S8 and Figure S1.

## Data Availability

• Considering the rarity of BID and that participants were recruited from a relatively limited geographic area, any information that could identify participants has been removed from the openly available dataset and materials. This dataset has been deposited at Mendeley Data. DOI is listed in the key resources table. • The code for replication of all analysis has been deposited at Mendeley Data and is publicly available as of the date of publication. DOI is listed in the key resources table. • Any additional information required to reanalyze the data reported in this paper is available from the lead contact upon request.

## References

[1] Barsalou L.W. (2008). Grounded Cognition. Annu. Rev. Psychol..

[2] Buccino G., Colagè I., Gobbi N., Bonaccorso G. (2016). Grounding meaning in experience: A broad perspective on embodied language. Neurosci. Biobehav. Rev..

[3] Nathan M.J., Schenck K.E., Vinsonhaler R., Michaelis J.E., Swart M.I., Walkington C. (2021). Embodied geometric reasoning: Dynamic gestures during intuition, insight, and proof. J. Educ. Psychol..

[4] Arminjon M., Preissmann D., Chmetz F., Duraku A., Ansermet F., Magistretti P.J. (2015). Embodied memory: unconscious smiling modulates emotional evaluation of episodic memories. Front. Psychol..

[5] Stel M., Van Knippenberg A. (2008). The Role of Facial Mimicry in the Recognition of Affect. Psychol. Sci..

[6] Berlucchi G., Aglioti S. (1997). The body in the brain: neural bases of corporeal awareness. Trends Neurosci..

[7] Blanke O. (2012). Multisensory brain mechanisms of bodily self-consciousness. Nat. Rev. Neurosci..

[8] Ehrsson H.H., Sathian K., Ramachandran V.S. (2020). Multisensory Perception.

[9] Berlucchi G., Aglioti S.M. (2010). The body in the brain revisited. Exp. Brain Res..

[10] Banakou D., Kishore S., Slater M. (2018). Virtually Being Einstein Results in an Improvement in Cognitive Task Performance and a Decrease in Age Bias. Front. Psychol..

[11] Gonzalez-Liencres C., Zapata L.E., Iruretagoyena G., Seinfeld S., Perez-Mendez L., Arroyo-Palacios J., Borland D., Slater M., Sanchez-Vives M.V. (2020). Being the Victim of Intimate Partner Violence in Virtual Reality: First- Versus Third-Person Perspective. Front. Psychol..

[12] Peck T.C., Seinfeld S., Aglioti S.M., Slater M. (2013). Putting yourself in the skin of a black avatar reduces implicit racial bias. Conscious. Cogn..

[13] Dijkerman C., Lenggenhager B. (2018). The body and cognition: The relation between body representations and higher level cognitive and social processes | Elsevier Enhanced Reader. Cortex.

[14] Damasio A.R., Everitt B.J., Bishop D. (1996). The Somatic Marker Hypothesis and the Possible Functions of the Prefrontal Cortex. Philos. Trans. R. Soc. Lond. B Biol. Sci..

[15] Bechara A., Damasio A.R. (2005). The somatic marker hypothesis: A neural theory of economic decision. Games Econ. Behav..

[16] Bolt E., Ho J.T., Roel Lesur M., Soutschek A., Tobler P.N., Lenggenhager B. (2021). Effects of a virtual gender swap on social and temporal decision-making. Sci. Rep..

[17] Lee J.J., Hardin A.E., Parmar B., Gino F. (2019). The Interpersonal Costs of Dishonesty: How Dishonest Behavior Reduces Individuals’ Ability to Read Others’ Emotions. J. Exp. Psychol. Gen..

[18] (2021). Commission to the Council and the European Parliament.

[19] Nowak M.A., Page K.M., Sigmund K. (2000). Fairness Versus Reason in the Ultimatum Game. Science.

[20] Lenggenhager B., Azevedo R.T., Mancini A., Aglioti S.M. (2013). Listening to your heart and feeling yourself: effects of exposure to interoceptive signals during the ultimatum game. Exp. Brain Res..

[21] Mancini A., Betti V., Panasiti M.S., Pavone E.F., Aglioti S.M. (2011). Suffering makes you egoist: Acute pain increases acceptance rates and reduces fairness during a bilateral ultimatum game. PLoS One.

[22] Vicario C.M., Kuran K.A., Rogers R., Rafal R.D. (2018). The effect of hunger and satiety in the judgment of ethical violations. Brain Cogn..

[23] Williams E.F., Pizarro D., Ariely D., Weinberg J.D. (2016). The Valjean effect: Visceral states and cheating. Emotion.

[24] Paulus M.P. (2007). Decision-making dysfunctions in psychiatry--altered homeostatic processing?. Science.

[25] Paulus M.P. (2007). Neural basis of reward and craving —a homeostatic point of view. Dialogues Clin. Neurosci..

[26] Scattolin M., Panasiti M.S., Aglioti S.M. (2022). Morality in the flesh: on the link between bodily self-consciousness, moral identity and (dis)honest behaviour. R. Soc. Open Sci..

[27] Scattolin M., Panasiti M.S., Villa R., Aglioti S.M. (2022). Reduced ownership over a virtual body modulates dishonesty. iScience.

[28] Lee S.W.S., Schwarz N. (2020). Grounded procedures: A proximate mechanism for the psychology of cleansing and other physical actions. Behav. Brain Sci..

[29] Scattolin M., Panasiti M.S., Aglioti S.M. (2021). Body ownership as a proxy for individual and social separation and connection. Behav. Brain Sci..

[30] Li X., Wei L., Soman D. (2010). Sealing the Emotions genie: The Effects of Physical Enclosure on Psychological Closure. Psychol. Sci..

[31] Frewen P., Lanius R. (2015).

[32] World Health Organization (2019). International statistical classification of diseases and related health problems.

[33] First M.B., Fisher C.E. (2012). Body Integrity Identity Disorder: The Persistent Desire to Acquire a Physical Disability. Psychopathology.

[34] First M.B. (2005). Desire for amputation of a limb: paraphilia, psychosis, or a new type of identity disorder. Psychol. Med..

[35] Weijs M.L., Ho J.T., Roel Lesur M., Lenggenhager B. (2022). Is this my foot? Experimentally induced disownership in individuals with body integrity dysphoria. Conscious. Cogn..

[36] De Vignemont F. (2015). Pain and Bodily Care: Whose Body Matters?. Australas. J. Philos..

[37] Moseley G.L., Gallace A., Spence C. (2012). Bodily illusions in health and disease: Physiological and clinical perspectives and the concept of a cortical ‘body matrix. Neurosci. Biobehav. Rev..

[38] Gandola M., Zapparoli L., Saetta G., Reverberi C., Salvato G., Squarza S.A.C., Invernizzi P., Sberna M., Brugger P., Bottini G., Paulesu E. (2021). Brain Abnormalities in Individuals with a Desire for a Healthy Limb Amputation: Somatosensory, Motoric or Both? A Task-Based fMRI Verdict. Brain Sci..

[39] Hänggi J., Vitacco D.A., Hilti L.M., Luechinger R., Kraemer B., Brugger P. (2017). Structural and functional hyperconnectivity within the sensorimotor system in xenomelia. Brain Behav..

[40] Hilti L.M., Hänggi J., Vitacco D.A., Kraemer B., Palla A., Luechinger R., Jäncke L., Brugger P. (2013). The desire for healthy limb amputation: structural brain correlates and clinical features of xenomelia. Brain.

[41] McGeoch P.D., Brang D., Song T., Lee R.R., Huang M., Ramachandran V.S. (2011). Xenomelia: a new right parietal lobe syndrome. J. Neurol. Neurosurg. Psychiatry.

[42] Oddo-Sommerfeld S., Hänggi J., Coletta L., Skoruppa S., Thiel A., Stirn A.V. (2018). Brain activity elicited by viewing pictures of the own virtually amputated body predicts xenomelia. Neuropsychologia.

[43] Saetta G., Hänggi J., Gandola M., Zapparoli L., Salvato G., Berlingeri M., Sberna M., Paulesu E., Bottini G., Brugger P. (2020). Neural Correlates of Body Integrity Dysphoria. Curr. Biol..

[44] Saetta G., Ruddy K., Zapparoli L., Gandola M., Salvato G., Sberna M., Bottini G., Brugger P., Lenggenhager B. (2022). White matter abnormalities in the amputation variant of body integrity dysphoria. Cortex.

[45] van Dijk M.T., van Wingen G.A., van Lammeren A., Blom R.M., de Kwaasteniet B.P., Scholte H.S., Denys D. (2013). Neural Basis of Limb Ownership in Individuals with Body Integrity Identity Disorder. PLoS One.

[46] Azevedo R.T., Panasiti M.S., Maglio R., Aglioti S.M. (2018). Perceived warmth and competence of others shape voluntary deceptive behaviour in a morally relevant setting. Br. J. Psychol..

[47] Panasiti M.S., Pavone E.F., Merla A., Aglioti S.M. (2011). Situational and dispositional determinants of intentional deceiving. PLoS One.

[48] Panasiti M.S., Pavone E.F., Mancini A., Merla A., Grisoni L., Aglioti S.M. (2014). The motor cost of telling lies: Electrocortical signatures and personality foundations of spontaneous deception. Soc. Neurosci..

[49] Panasiti M.S., Cardone D., Pavone E.F., Mancini A., Merla A., Aglioti S.M. (2016). Thermal signatures of voluntary deception in ecological conditions. Sci. Rep..

[50] Schepisi M., Porciello G., Aglioti S.M., Panasiti M.S. (2020). Oculomotor behavior tracks the effect of ideological priming on deception. Sci. Rep..

[51] Aoyama A., Krummenacher P., Palla A., Hilti L.M., Brugger P. (2012). Impaired Spatial-Temporal Integration of Touch in Xenomelia (Body Integrity Identity Disorder). Spat. Cogn. Comput..

[52] Ho J.T., Krummenacher P., Lenggenhager B. (2022). Not my body, not my pain? Pain perception and placebo analgesia in individuals with body integrity dysphoria. Cortex.

[53] Lenggenhager B., Hilti L., Brugger P. (2015). Disturbed body integrity and the “rubber foot illusion. Neuropsychology.

[54] Stone K.D., Dijkerman H.C., Bekrater-Bodmann R., Keizer A. (2019). Mental rotation of feet in individuals with body integrity identity disorder, lower-limb amputees, and normally-limbed controls. PLoS One.

[55] Stone K.D., Kornblad C.A.E., Engel M.M., Dijkerman H.C., Blom R.M., Keizer A. (2020). An Investigation of Lower Limb Representations Underlying Vision, Touch, and Proprioception in Body Integrity Identity Disorder. Front. Psychiatry.

[56] Stone K.D., Kornblad C.A.E., Engel M.M., Dijkerman H.C., Blom R.M., Keizer A. (2021). Lower limb peripersonal space and the desire to amputate a leg. Psychol. Res..

[57] Eppinger B., Schuck N.W., Nystrom L.E., Cohen J.D. (2013). Reduced Striatal responses to reward prediction errors in older compared with younger adults. J. Neurosci..

[58] Eppinger B., Nystrom L.E., Cohen J.D. (2012). Reduced sensitivity to immediate reward during decision-making in older than younger adults. PLoS One.

[59] Gerlach P., Teodorescu K., Hertwig R. (2019). The truth about lies: A meta-analysis on dishonest behavior. Psychol. Bull..

[60] Haggard P. (2017). Sense of agency in the human brain. Nat. Rev. Neurosci..

[61] Coren S. (1993). The lateral preference inventory for measurement of handedness, footedness, eyedness, and earedness: Norms for young adults. Bull. Psychon. Soc..

[62] Büsch D., Hagemann N., Bender N. (2009). Das lateral preference inventory: Itemhomogenität der deutschen version. Z. für Sportpsychol..

[63] Mazar N., Amir O., Ariely D. (2008). The dishonesty of honest people: A theory of self-concept maintenance. J. Mark. Res..

[64] Zhong C.-B., Liljenquist K. (2006). Washing away your sins: Threatened morality and physical cleansing. Science.

[65] Slater M., Perez-Marcos D., Ehrsson H.H., Sanchez-Vives M.V. (2009). Inducing illusory ownership of a virtual body. Front. Neurosci..

[66] Porciello G., Bufalari I., Minio-Paluello I., Di Pace E., Aglioti S.M. (2018). The “Enfacement” illusion: A window on the plasticity of the self. Cortex.

[67] Botvinick M., Cohen J. (1998). Rubber hands ‘feel’ touch that eyes see. Nature.

[68] Villa R., Ponsi G., Scattolin M., Panasiti M.S., Aglioti S.M. (2022). Social, affective, and non-motoric bodily cues to the Sense of Agency: A systematic review of the experience of control. Neurosci. Biobehav. Rev..

[69] Kilteni K., Groten R., Slater M. (2012). The Sense of Embodiment in Virtual Reality. Presence. (Camb)..

[70] Haggard P., Clark S., Kalogeras J. (2002). Voluntary action and conscious awareness. Nat. Neurosci..

[71] Blakemore S.-J., Wolpert D., Frith C. (2000). Why can’t you tickle yourself?. Neuroreport.

[72] Tsakiris M., Schütz-Bosbach S., Gallagher S. (2007). On agency and body-ownership: Phenomenological and neurocognitive reflections. Conscious. Cogn..

[73] Gallagher I. (2000). Philosophical conceptions of the self: implications for cognitive science. Trends Cogn. Sci..

[74] de Haan S., de Bruin L. (2010). Reconstructing the minimal self, or how to make sense of agency and ownership. Phenomenol. Cogn. Sci..

[75] Stoet G. (2017). PsyToolkit: A novel web-based method for running online questionnaires and reaction-time experiments. Teach. Psychol..

[76] R Core Team (2022). R: A Language and Environment for Statistical Computing.

[77] Posit team (2022). RStudio. Integrated Development Environment for R.

[78] Adler N.E., Epel E.S., Castellazzo G., Ickovics J.R. (2000). Relationship of subjective and objective social status with psychological and physiological functioning: preliminary data in healthy, white women. Health Psychol..

[79] Ostrove J.M., Adler N.E., Kuppermann M., Washington A.E. (2000). Objective and subjective assessments of socioeconomic status and their relationship to self-rated health in an ethnically diverse sample of pregnant women. Health Psychol..

[80] Brown V.A. (2021). An Introduction to Linear Mixed-Effects Modeling in R. Adv. Methods Pract. Psychol. Sci..

