# Supplementary material for: Ownership of the affected leg is further reduced following deceptive behaviors in body integrity dysphoria
Source: iScience. 2023 Aug 7;26(9):107551. doi: 10.1016/j.isci.2023.107551 (PMC10469995; doi:10.1016/j.isci.2023.107551)
Supplement: Document S1. Figures S1 and Tables S1–S8 [file mmc1.pdf]

iScience, Volume *26*

Supplemental information

Ownership of the affected leg is further reduced following deceptive behaviors in body integrity dysphoria

Marina Scattolin, Maria Serena Panasiti, Jasmine T. Ho, Bigna Lenggenhager, and Salvatore Maria Aglioti

Table S1. Hand and foot preferences of participants in the two experimental groups (related to sections *Results* and *STAR Methods – Method details*). CG and BID refer to the control group and Body Integrity Dysphoria group, respectively. 'No.' indicates the number of occurrences for each entry; '%' indicates the corresponding percentage of occurrences within each group.

**Lateral preference**	**No.<br>CG group**	**%<br>CG group**	**No.<br>BID group**	**%<br>BID group**
**Hand preference**				
Left	2	11.76	4	20
No preference	0	0	0	0
Right	15	88.24	16	80
**Foot preference**				
Left	3	17.65	3	15
No preference	0	0	1	5
Right	14	82.35	16	80

Table S2. Results of the linear mixed model to predict baseline SoO ratings (related to section *Results – SoO ratings*). Column β contains the beta coefficient associated with each fixed predictor; "Lower CI" and "Upper CI" refer to the 2.5% and 97.5% Confidence Interval, respectively; t indicates the t-statistics; column p presents the p-value associated with each coefficient. Finally, Std β reports standardized beta coefficients.

**Fixed predictor**	**β**	**Lower CI**	**Upper CI**	***t*(68)**	***p***	**Std β**
Intercept	97.00	88.80	105.20	23.60	< .001	0.52
Group	-59.05	-70.20	-47.90	-10.56	< .001	-1.90
Leg condition	0.24	-10.16	10.63	0.05	.964	7.57e-03
Group * Leg condition	57.86	43.73	72.00	8.17	< .001	1.86

Table S3. Results of the linear mixed model to predict baseline SoA ratings (related to section *Results – SoA ratings*). Column β contains the beta coefficient associated with each fixed predictor; "Lower CI" and "Upper CI" refer to the 2.5% and 97.5% Confidence Interval, respectively; t indicates the t-statistics; column p presents the p-value associated with each coefficient. Finally, Std β reports standardized beta coefficients.

Fixed predictor	β	Lower CI	Upper CI	*t*(68)	*p*	Std β
Intercept	93.94	84.48	103.40	19.82	< .001	0.23
Group	-21.89	-34.75	-9.03	-3.40	.001	-1.00
Leg condition	1.29	-10.27	12.86	0.22	.825	0.06
Group * Leg condition	23.21	7.48	38.94	2.94	.006	1.06

Table S4. Results of the logistic mixed model to predict participants' behavior during the U-TLCG (related to section *Results – Leg ownership does not affect moral decisions*). Column β contains the beta coefficient associated with each fixed predictor; "Lower CI" and "Upper CI" refer to the 2.5% and 97.5% Confidence Interval, respectively; z indicates the z value; column p presents the p-value associated with each coefficient. Finally, Std β reports standardized beta coefficients.

Fixed predictor	β	Lower CI	Upper CI	*z*	*p*	Std β
Intercept	-0.51	-2.06	1.03	-0.65	.517	-0.52
Situation	-2.48	-4.50	-0.46	-2.41	.016	-2.47
Leg condition	0.24	-0.44	0.93	0.69	.489	0.24
Group	1.97	-0.17	4.12	1.80	.071	2.00
Involvement ratings	-0.10	-0.77	0.58	-0.28	.781	-0.09
Baseline SoA ratings	-0.01	-0.03	0.00	-1.39	.164	-0.23
Situation * Leg condition	-0.28	-1.35	0.80	-0.51	.613	-0.28
Situation * Group	-2.87	-5.69	-0.05	-1.99	.046	-2.90
Leg condition * Group	0.18	-0.85	1.22	0.35	.729	0.17
Situation * Leg condition * Group	0.23	-1.36	1.81	0.28	.779	0.25

Table S5. Results of the linear mixed model to predict the change of SoO ratings related to self-gain lies (related to section *Results - Self-gain lies are followed by further reductions of leg ownership*). Column β contains the beta coefficient associated with each fixed predictor; "Lower CI" and "Upper CI" refer to the 2.5% and 97.5% Confidence Interval, respectively; t indicates the t-statistics; column p presents the p-value associated with each coefficient. Finally, Std β reports standardized beta coefficients.

Fixed predictor	β	Lower CI	Upper CI	*t*(62)	*p*	Std β
Intercept	-0.65	-6.86	5.56	-0.21	.835	0.12
Group	9.69	-0.63	20.00	1.88	.065	-0.25
Leg condition	0.87	-8.22	9.96	0.19	.850	-5.89e-03
Self-gain lies (%)	2.22	-9.67	14.12	0.37	.710	0.09
Change of SoA	0.09	-0.25	0.43	0.54	.592	0.06
Involvement ratings	-2.38	-4.68	-0.08	-2.07	.043	-0.25
Group * Leg condition	-12.36	-26.87	2.16	-1.70	.094	0.25
Group * Self-gain lies (%)	-21.61	-37.60	-5.63	-2.70	.009	-0.85
Leg condition * Self-gain lies (%)	-1.64	-17.69	14.41	-0.20	.839	-0.06
Group * Leg condition * Self-gain lies (%)	26.33	4.54	48.13	2.42	.019	1.04

Table S6. Results of the linear mixed model to predict the change of SoO ratings related to other-gain lies (related to section *Results - Other-gain lies do not modulate leg ownership*). Column β contains the beta coefficient associated with each fixed predictor; “Lower CI” and “Upper CI” refer to the 2.5% and 97.5% Confidence Interval, respectively; t indicates the t-statistics; column p presents the p-value associated with each coefficient. Finally, Std β reports standardized beta coefficients.

Fixed predictor	β	Lower CI	Upper CI	*t*(62)	*p*	Std β
Intercept	0.85	-5.09	6.78	0.28	.777	0.08
Group	-4.57	-12.72	3.59	-1.12	.268	-0.42
Leg condition	-0.38	-8.49	7.72	-0.09	.925	0.03
Other-gain lies (%)	-4.59	-30.45	21.27	-0.35	.724	-0.09
Change of SoA	0.11	-0.26	0.49	0.61	.541	0.07
Involvement ratings	-1.77	-4.28	0.75	-1.41	.165	-0.18
Group * Leg condition	6.01	-4.96	16.98	-1.09	.278	0.48
Group * Other-gain lies (%)	3.23	-44.28	50.73	0.14	.892	0.06
Leg condition * Other-gain lies (%)	5.20	-26.97	37.36	0.32	.748	0.10
Group * Leg condition * Other-gain lies (%)	-10.02	-63.69	43.65	-0.37	.710	-0.20

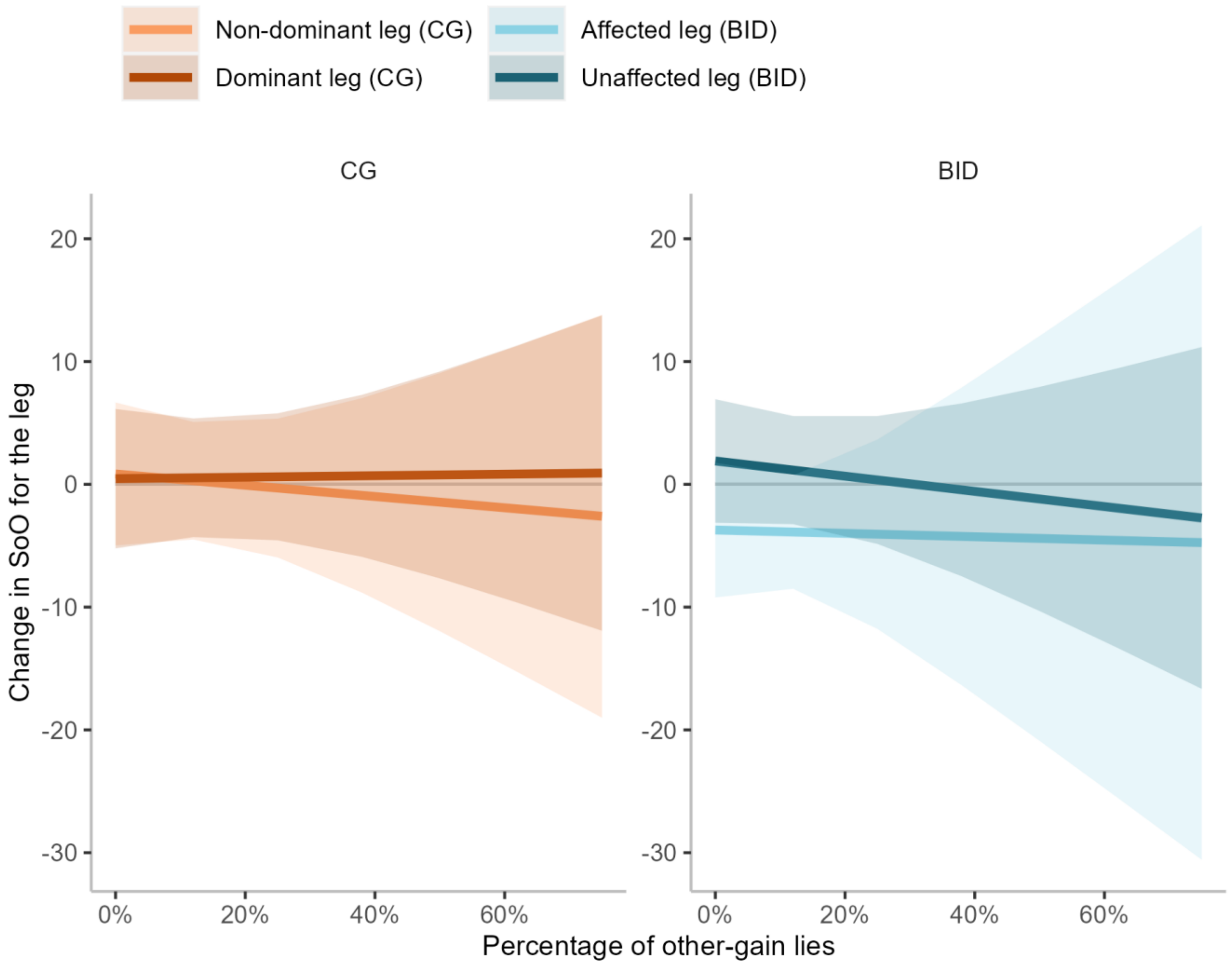

Figure S1. Change in the sense of ownership (SoO) after the Unfair Temptation to Lied Card Game (U-TLCG) as a function of group, leg condition and the percentage of other-gain lies *(related to section* Results - *Other-gain lies do not modulate leg ownership)*. The leftmost panel represents the change of SoO ratings for participants in the control group (CG), while ratings of participants in the Body Integrity Dysphoria (BID) group are displayed in the rightmost panel. The plot shows regression lines for each leg. The horizontal line at 0 indicates the absence of difference between the two assessments. Values above the horizontal line indicate that SoO ratings are higher following the U-TLCG. Values below the horizontal line indicate that SoO ratings were lower following the U-TLCG. The shaded bands represent 95% confidence intervals.

Table S7. Results of the linear mixed model to predict the change of SoA ratings related to self-gain lies ($R^2_{marginal}$ = 0.03, $R^2_{conditional}$ = 0.14). The table refers to section *Results - Leg agency is not affected by self- nor other-gain lies*. Column β contains the beta coefficient associated with each fixed predictor; "Lower CI" and "Upper CI" refer to the 2.5% and 97.5% Confidence Interval, respectively; t indicates the t-statistics; column p presents the p-value associated with each coefficient. Finally, Std β reports standardized beta coefficients.

Fixed predictor	β	Lower CI	Upper CI	*t*(62)	*p*	Std β
Intercept	-0.38	-4.95	4.20	-0.17	.869	0.09
Group	-0.41	-8.21	7.40	-0.10	.917	-0.24
Leg condition	1.30	-5.52	8.13	0.38	.704	0.06
Self-gain lies (%)	2.34	-6.42	11.11	0.53	.595	0.14
Change of SoO	0.05	-0.14	0.23	0.52	.602	0.07
Involvement ratings	-0.47	-2.19	1.24	-0.55	.585	-0.08
Group * Leg condition	-1.54	-12.70	9.62	-0.28	.784	0.06
Group * Self-gain lies (%)	-1.96	-14.39	10.48	-0.31	.754	-0.12
Leg condition * Self-gain lies (%)	-1.61	-13.66	10.44	-0.27	.790	-0.10
Group * Leg condition * Self-gain lies (%)	3.38	-13.71	20.46	0.40	.694	0.21

Table S8. Results of the linear mixed model to predict the change of SoA ratings related to other-gain lies ($R^2_{marginal}$ = 0.03, $R^2_{conditional}$ = 0.14). The table refers to section *Results - Leg agency is not affected by self- nor other-gain lies*. Column β contains the beta coefficient associated with each fixed predictor; "Lower CI" and "Upper CI" refer to the 2.5% and 97.5% Confidence Interval, respectively; t indicates the t-statistics; column p presents the p-value associated with each coefficient. Finally, Std β reports standardized beta coefficients.

Fixed predictor	β	Lower CI	Upper CI	*t*(62)	*p*	Std β
Intercept	0.06	-3.91	4.03	0.03	.976	0.01
Group	0.05	-5.44	5.55	0.02	.984	-0.19
Leg condition	1.21	-4.05	6.46	0.46	.648	0.13
Other-gain lies (%)	3.26	-13.96	20.49	0.38	.706	0.10
Change of SoO	0.05	-0.11	0.22	0.64	.524	0.08
Involvement ratings	-0.36	-2.10	1.39	-0.41	.683	-0.06
Group * Leg condition	-0.69	-7.88	6.49	-0.19	.848	0.06
Group * Other-gain lies (%)	-10.53	-42.07	21.00	-0.67	.507	-0.33
Leg condition * Other-gain lies (%)	-3.09	-24.10	17.93	-0.29	.770	-0.10
Group * Leg condition * Other-gain lies (%)	8.89	-26.17	43.95	0.51	.614	0.27

Table S9. Anamnestic information of participants in the Control Group (CG) and Body Integrity Dysphoria (BID) group, respectively (related to section *STAR Methods - Experimental model and subject details*). ‘No.’ indicates the number of occurrences for each entry; ‘%’ indicates the corresponding percentage of occurrences within each group.

Anamnestic information	No. CG group	% CG group	No. BID group	% BID group
**Psychiatric conditions**	**1**	**5.88**	**2**	**10**
Depression	1	5.88	1	5
Burnout	0	0	1	5
**Psychiatric Medications**				
Antidepressants	1	5.88	1	5
**Other Medications**	**6**	**35.29**	**7**	**35**
Hormone Replacement Therapy	0	0	1	5
Heart medication	1	5.88	0	0
Hypertension treatment	3	17.65	3	15
Diabetes treatment	1	5.88	0	0
Muscular pain medication	1	5.88	0	0
Hormonal therapy for hypo- and hyper-thyroidism	0	0	2	10
Menopausal symptoms treatment	0	0	1	5
